# Multiobjective de novo drug design with recurrent neural networks and nondominated sorting

**DOI:** 10.1186/s13321-020-00419-6

**Published:** 2020-02-18

**Authors:** Jacob Yasonik

**Affiliations:** Homestead High School, Mequon, WI 53092 USA

**Keywords:** Deep learning, Multiobjective optimization, De novo drug design

## Abstract

Research productivity in the pharmaceutical industry has declined significantly in recent decades, with higher costs, longer timelines, and lower success rates of drug candidates in clinical trials. This has prioritized the scalability and multiobjectivity of drug discovery and design. De novo drug design has emerged as a promising approach; molecules are generated from scratch, thus reducing the reliance on trial and error and premade molecular repositories. However, optimizing for molecular traits remains challenging, impeding the implementation of de novo methods. In this work, we propose a de novo approach capable of optimizing multiple traits collectively. A recurrent neural network was used to generate molecules which were then ranked based on multiple properties by a nondominated sorting algorithm. The best of the molecules generated were selected and used to fine-tune the recurrent neural network through transfer learning, creating a cycle that mimics the traditional design–synthesis–test cycle. We demonstrate the efficacy of this approach through a proof of concept, optimizing for constraints on molecular weight, octanol-water partition coefficient, the number of rotatable bonds, hydrogen bond donors, and hydrogen bond acceptors simultaneously. Analysis of the molecules generated after five iterations of the cycle revealed a 14-fold improvement in the quality of generated molecules, along with improvements to the accuracy of the recurrent neural network and the structural diversity of the molecules generated. This cycle notably does not require large amounts of training data nor any handwritten scoring functions. Altogether, this approach uniquely combines scalable generation with multiobjective optimization of molecules.

## Introduction

Drug discovery is the first step in the drug development pipeline and aims to identify drug candidates for further study in clinical trials [1]. Yet despite many technological advances, productivity has declined. Research and development (R&D) costs have doubled nearly every nine years since 1950: an 80-fold increase when accounting for inflation [2]. Concerns over the scalability of current methods have arisen given their reliance on trial and error. The primary methods of drug discovery, high throughput screening (HTS) and virtual screening (VS), evaluate molecules in predefined repositories to identify promising leads [3, 4]. However, the sheer magnitude of the chemical search space makes such systems, operating alone, impractical in larger experiments. Recent estimates have deemed 10^60^ drug-like molecules as synthetically accessible [5]. Additional challenges have surfaced in the paltry success rates of lead molecules in clinical trials. Across all medicinal groups, just 13.8% of leads make it past the first stage of clinical trials; oncology has the lowest success rate at 3.4% [6]. Candidate molecules are failing to meet basic physicochemical criteria of pharmaceutical drugs [7]. These inefficiencies inspire a need for a scalable and multiobjective approach to drug discovery.

A promising, scalable method of drug discovery has emerged in de novo drug design. By generating molecules from scratch, potentially vastly different from those in available molecular repositories, de novo drug design can better represent the entire chemical space [8]. Machine learning has been increasingly applied with successes in generating synthetically reasonable molecules [9]. However, a complete system able to both generate valid molecules and optimize multiple traits has remained elusive. Autoencoders have been used to encode molecules into a continuous vector space; in principle, this makes for easy optimization [10–12]. Encoding inherently discrete molecules into continuous space poses intuitive challenges though. Generated molecules are often synthetically unreasonable. Evolutionary algorithms also struggle to generate valid molecules but yield promising results in optimization [13]. A large variety of evolutionary selection mechanisms have proven successful in other multiobjective optimization problems [14–17] and show promise in drug discovery. Recurrent neural networks have been successful in generating reasonable molecules through an approach based on natural language processing. Molecules are encoded as strings using the Simplified Molecular Input Line-Entry System (SMILES) [18–20]. The recurrent neural network is then trained to predict the next SMILES character given a sequence of previous characters. Accuracies of valid molecules nearing 90% have been achieved through this method [21–24]. Generative adversarial networks (GANs) using a recurrent neural network as the generative network have also shown promise [25]. These recent successes in generating valid molecules with recurrent neural networks have now shifted attention to optimizing molecular properties. Reinforcement learning has been used, though handwritten reward functions can be exploited by the network through trivial solutions that seemingly fit the parameters [26]. Ideally, a system of de novo drug design would be able to take cues from evolutionary algorithms in multiobjective optimization while still generating reasonable molecules.

**Fig. 1:**
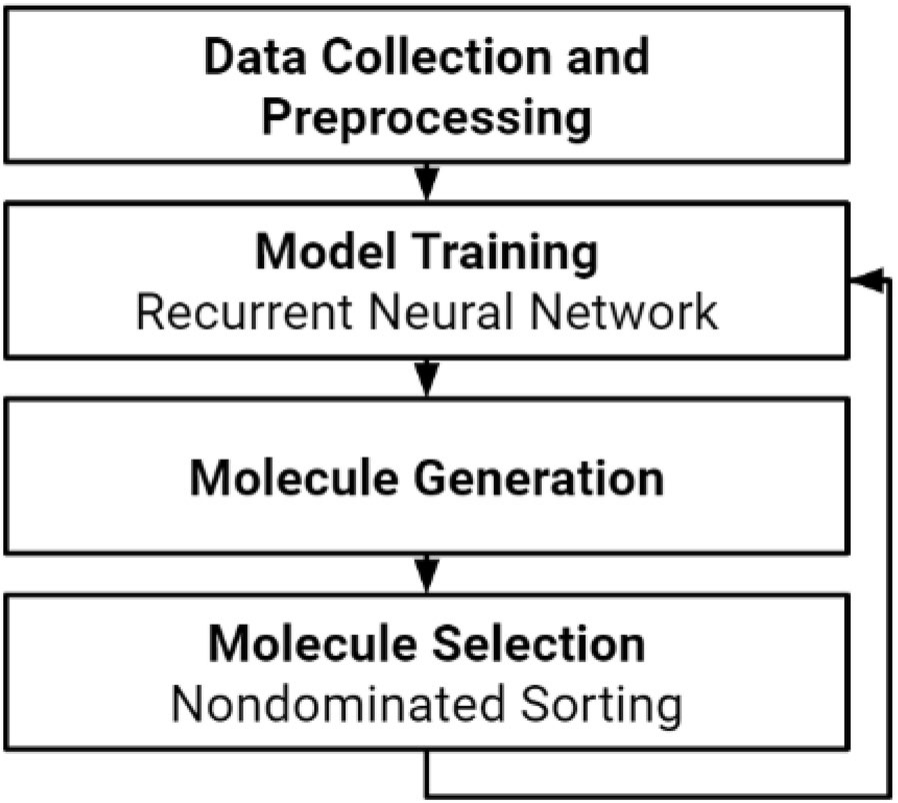
Schema of the proposed de novo drug design cycle

In this work, we propose a multiobjective, evolutionary de novo drug design approach (Fig. 1). A recurrent neural network is used to generate molecules, and the best are selected and used to retrain the network through transfer learning. Transfer learning allows knowledge to be transferred between tasks, and has proven to be an efficient way of improving the accuracy of models on narrowly-defined tasks [27–29]. The best of the generated molecules are selected by the novel application of a nondominated sorting algorithm, a proven method of multiobjective optimization. We optimize five different criteria of drug candidates that stem from the Rule of Three, an extension of the Lipinski Rule of Five [30, 31]. Such guidelines are commonly used as preliminary tests to evaluate fragments, lead compounds, and drug-like molecules [32]. We optimize these properties as a proof of concept to validate the unique multiobjectivity of this approach to de novo drug design.

## Methods

### Data collection and preprocessing

**Fig. 2:**
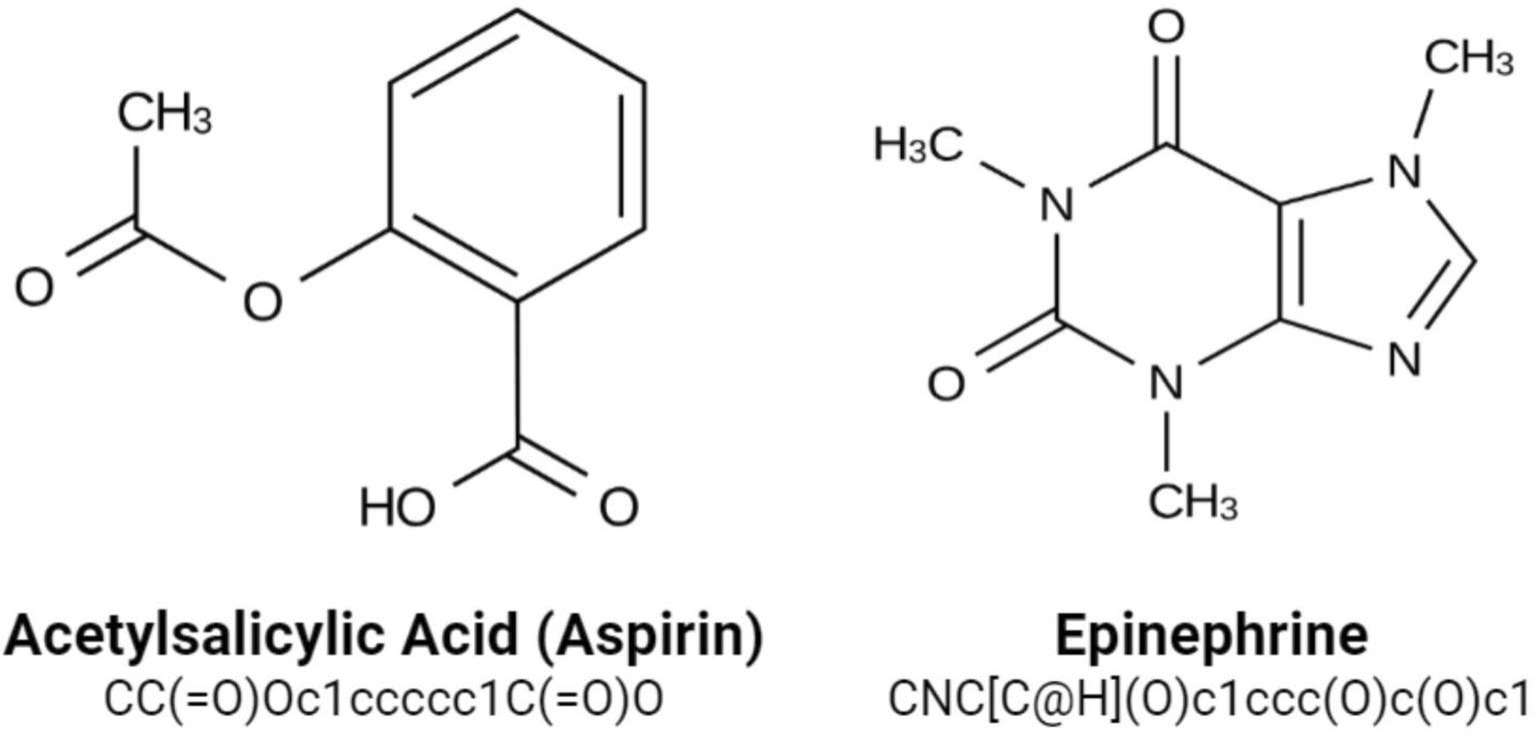
Example SMILES notations for various molecules

A training dataset of 500,000 molecules was assembled from the open-source ChEMBL dataset of drug-like molecules, curated by the European Bioinformatics Institute [33]. Molecules were represented using the SMILES string notation for easy interpretation by the recurrent neural network model we employ. SMILES was specifically designed with grammatical consistency and machine friendliness in mind, using characters to represent atoms, bonds, and chemical structures (Fig. 2) [20]. For example, aromatic and aliphatic carbon atoms are represented by the symbols c and C. Single, double, and triple bonds are represented by the characters -, = , #, respectively. Parenthese enclosures are used to show branches, and rings are indicated by digits immediately following the atoms where the ring is closed. The 500,000 molecules collected totalled 25 million SMILES characters. Additionally, start and end characters of “G” (go) and “\n” (new line) were appended to each molecule, yielding a total vocabulary of 53 unique characters within the dataset. All molecules were between 35 and 75 characters in length. A one-hot encoding was applied to these SMILES molecules such that each SMILES character was represented by a 53 dimensional vector of zeros with a one in the appropriate index of the character. This data was then used to train a recurrent neural network to generate valid molecules.

## Recurrent neural networks

Recurrent neural networks (RNNs) have proven successful in modeling sequential data, commonly found in the form of natural language processing. In addition to capturing the grammatical structure of the data, recurrent neural networks are able to interpret its meaning as well [34]. Abstractly, recurrent neural networks can be considered as many copies of the same neural network, each passing data to its successor through a hidden state. Each neural network assigns a probability to the next element in the sequence given all those that came before it by factoring in this hidden state. It follows that, given network parameters *θ*, the probability of the entire sequence ${\varvec{S}}={\mathrm{s}}_{1}\dots {\mathrm{s}}_{\mathrm{t}}$ of size ${\varvec{t}}$ time steps is:

$${P}_{\uptheta }\left({\varvec{S}}\right)= {P}_{\theta }({{\varvec{s}}}_{1}){P}_{\theta }({{\varvec{s}}}_{2}|{{\varvec{s}}}_{1}){P}_{\theta }({{\varvec{s}}}_{3}|{{\varvec{s}}}_{1}{{\varvec{s}}}_{2})...{P}_{\theta }({{\varvec{s}}}_{{\varvec{t}}}|{{\varvec{s}}}_{1}...{{\varvec{s}}}_{{\varvec{t}}-1})$$

However, data can be diluted as it moves through the hidden state for a long time, resulting in the problem of long-term dependencies [35]. Specifically, gradients calculated during backpropagation in training may vanish or explode, preventing the network from capturing the data. This problem most clearly manifests itself in properly opening and closing parentheses. Vanilla (RNNs) often forget to close brackets due to the large gap between them. Modeling SMILES strings, as we do in this work, lends itself to this problem. Thus we use the long short term memory (LSTM) recurrent neural network.

The LSTM network is a type of recurrent neural network designed to accurately model long-term dependencies [36]. Unlike vanilla RNNs, LSTMs are composed of cells, each with three neural network layers called gates. The forget gate, update gate, and output gate determine what information to retain in an additional cell state. The cell state passes through the entire network; in this way, the hidden state of an LSTM acts as a short term memory, while the cell state acts as a long term memory. We trained an LSTM network on our dataset of SMILES molecules to generate new, valid molecules.

## Training the LSTM network

**Fig. 3:**
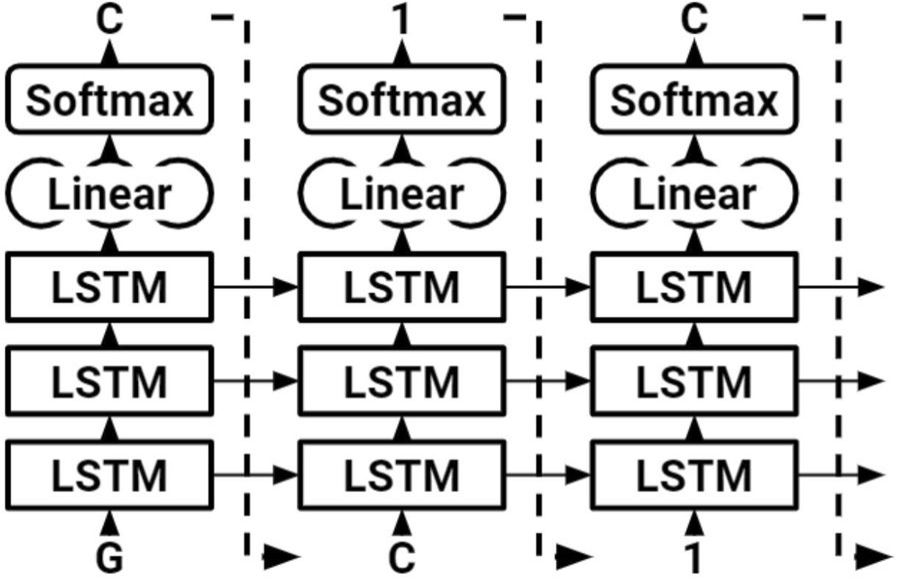
The LSTM used to generate SMILES strings. The character “G” is inputted to start, initializing the hidden and cell states. The network begins sampling symbol by symbol until the end character, “\n,” is produced

Our network was composed of three stacked LSTM layers, each of size 1024, regularized with a 0.2 dropout ratio (Fig. 3). This amounted to 21 million trainable parameters. Sequences 75 time steps in length were fed into the network in batches of size 128. A dense layer was applied after the LSTM cells to yield the output logits, which were then converted to probabilities by a Softmax layer during sampling. Backpropagation through time was used to train the network with the cross entropy loss function and ADAM optimizer [37, 38]. The model was created using the popular Python machine learning library Pytorch [39]. Molecules were sampled from the model during training to inspect progress (Table 1); the model quickly learns to generate valid molecules.

**Table 1:** Molecules sampled during training

Iteration	Generated example	Valid
0	LCtACFS5AF@t-rl(= sL#)	False
100	CN1C(=O)CCCNc2cccnc2N2CCN(C)3=O	False
500	CO1)C(= O)NC2CCN(CCN3[C@@H](C(=O)CCC4)c3c1CSC(= O)= C)C(= O)O	False
1000	CCCC=C(c1ccc(OCCCC(C)(C)C(=O)O)c(Sc2ccccc2)c1)C(=O)O	True
10,000	CC(C)(C)OC(=O)N[C@@H](Cc1c[nH]c2ccccc12)C(=O)N[C@@H](Cc3ccccc3)C(= O)N	True
100,000	COc1ccccc1NC(= S)NC2CC(C)(C)Oc3ccc(F)cc23	True

## Nondominated sorting

Optimizing many objectives poses a challenge in many fields. Criteria are frequently of a conflicting nature, making it difficult to measure and rank solutions let alone optimize them. Research in multiobjective optimization problems has shifted from trying to find a singular best solution to finding a set of Pareto optimal, or nondominated, solutions [14]. Nondominated sorting compares solutions in pairs; if solution *A* is better than or equal to solution *B* in all objectives measured, and *A* is better than *B* in at least one objective (i.e., the objective values are not all equal), then solution *A* is said to dominate solution *B*. Solutions that are not dominated by any other solution in the population are declared nondominated [15]. More formally, given a multiobjective problem to minimize objective vector ${\varvec{u}}, \mathrm{m}\mathrm{i}\mathrm{n}\{{\varvec{u}}=\left({\mathrm{u}}_{1},...,{\mathrm{u}}_{\mathrm{n}}\right)\}$, we have the following ranking rules:

I. Given two solution vectors ***u***^**1**^ and ***u***^**2**^, we say ${{\varvec{u}}}^{2}=\left({u}_{1}^{2}, . . . {,u}_{n}^{2}\right) \mathrm{i}\mathrm{s}$ superior to (dominates) solution vector ${{\varvec{u}}}^{1}=\left({u}_{1}^{1}, . . . {,u}_{n}^{1}\right) \mathrm{i}\mathrm{f}$ and only if ${{\varvec{u}}}^{2}$ is partially less than ${{\varvec{u}}}^{1}: \left({{\varvec{u}}}^{2} p<{\boldsymbol{ }{\varvec{u}}}^{1}\right).$ That is, $\forall i=1,. . . ,n,{ u}_{i}^{2}\le {u}_{i}^{1} \wedge \exists i=1,...,n : {u}_{i}^{2}<{u}_{u}^{1}$.
II. Solution vector ${{\varvec{u}}}^{2}$ is said to be inferior to (dominated by) solution vector ${{\varvec{u}}}^{1}$ if and only if vector ${{\varvec{u}}}^{1}$dominates ${{\varvec{u}}}^{2}$.
III. Solution vectors ${{\varvec{u}}}^{1}$ and ${{\varvec{u}}}^{2}$ are non-inferior to one another if and only if vector ${{\varvec{u}}}^{2}$ is neither superior nor inferior to vector ${{\varvec{u}}}^{1}$.

In this work, we used Fonseca and Fleming’s nondominated sorting algorithm [17] to compare molecules generated by the LSTM network based on the criteria outlined in the Rule of Three. Each solution (molecule) is ranked based on the number of solutions in the population by which it is dominated. Then nondominated solutions are not dominated by any other solutions and assigned rank zero. Dominated solutions are given values between $1$ and $\left({\varvec{k}}-1\right)$, where ${\varvec{k}}$ is the total number of solutions in the population, corresponding to how many other solutions they are inferior to. This algorithm was chosen for its simplicity and efficiency as a ranking method, having computational complexity ${\varvec{O}}\left({\mathrm{n}}^{2}\right)$. It follows that, in our implementation, nondominated molecules are the most optimal as per the constraints outlined, superior molecules are better than inferior molecules, and non-inferior molecules are tied.

## Transfer learning

Machine learning necessitates large quantities of data to train on, yet this is not always available: particularly in very narrowly-defined problems. Transfer learning has been applied successfully in such situations. In transfer learning, a model is trained on a source task and then retrained on a new, related task: the target task [29]. This requires less data to train on and has also been shown to result in significant improvements in accuracy [27]. We trained the LSTM network to generate valid molecules as a source task, and then retrained it to optimize specific properties as the target task. This process of generating valid molecules—selecting the best molecules—retraining the network simulates the traditional design–synthesis–test cycle far more rapidly.

## The rule of three

Early stage drug discovery necessitates quick evaluation of molecules to identify those suitable for further research. This has spurred the use of various multiobjective guidelines to estimate the potential of lead molecules, the most famous of which being Lipinski’s Rule of Five [30]. Many drug candidates do not subscribe to any such guidelines, and as such, many of the molecules used as training data from the ChEMBL training data do not align with their objectives. We apply these constraints solely as an approximation to assess the molecules generated by the LSTM model. Extensions to the Rule of Five have come about with varying degrees of accuracy [31, 32]. In this work, we use the Rule of Three (RO3), commonly applied to fragment-based lead discovery to identify promising lead compounds, to evaluate and optimize molecules generated by the LSTM network as a proof of concept. A compound subscribing to the RO3 is defined as having [31]:

- Octanol–water partition coefficient logP ≤ 3
- Molecular mass ≤ 300 daltons
-  ≤ 3 hydrogen bond donors
-  ≤ 3 hydrogen bond acceptors
-  ≤ 3 rotatable bonds

The open-source Python cheminformatics library RDKit was used to evaluate these properties in the molecules generated [40]. Molecular weight was considered instead of molecular mass as RDKit is currently unable to measure molecular mass directly. Other variants to the RO3, in particular, the Ghose Filter, set a limit on molecular weight instead of molecular mass [41]. The Ghose Filter confines the molecular weight to a maximum of 480 g/mol, which was used here. Thus we optimize for the following constraints:

- Octanol–water partition coefficient logP ≤ 3
- Molecular weight ≤ 480 g/mol
-  ≤ 3 hydrogen bond donors
-  ≤ 3 hydrogen bond acceptors
-  ≤ 3 rotatable bonds

## Results

### Generating molecules

One million SMILES characters were sampled from the LSTM network following training, yielding 19,722 molecules, none of which were in the original training data. RDKit was used to evaluate the molecules for validity and other properties [40]. Of the generated molecules, 77% were valid and 6,295 were duplicates. Filtering invalid and duplicated molecules left 9,415 unique, novel, and valid molecules.

**Fig. 4:**
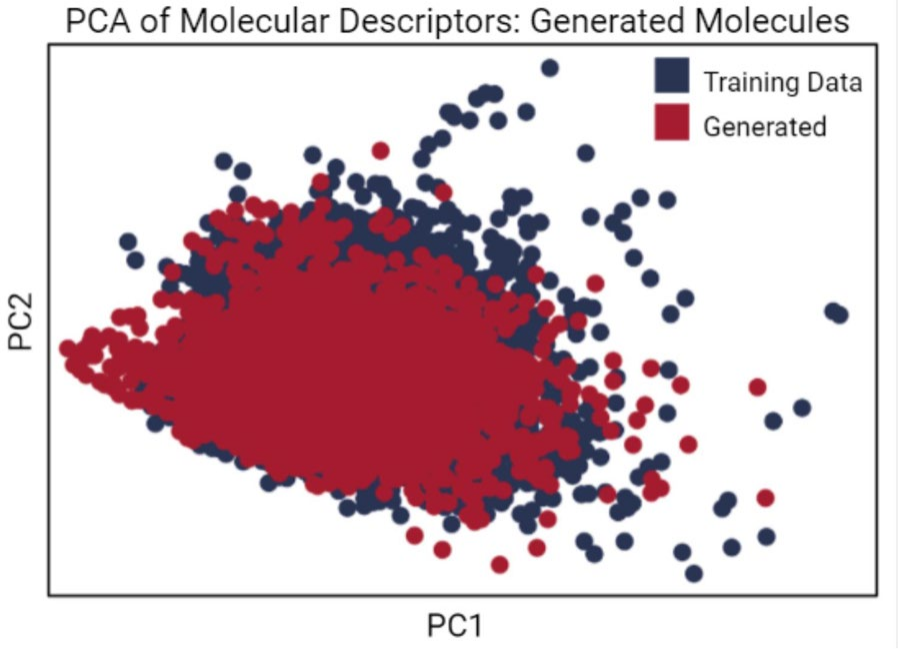
PCA projection of the molecular descriptors of molecules in the training data and molecules generated by the LSTM. Five molecular descriptors were evaluated for each of the molecules generated by the LSTM and 50,000 randomly selected molecules from the training data. Principal component analysis (PCA) was used for dimensionality reduction to plot the data, with generated molecules in red and training data molecules in blue. The distributions are closely aligned

**Fig. 5:**
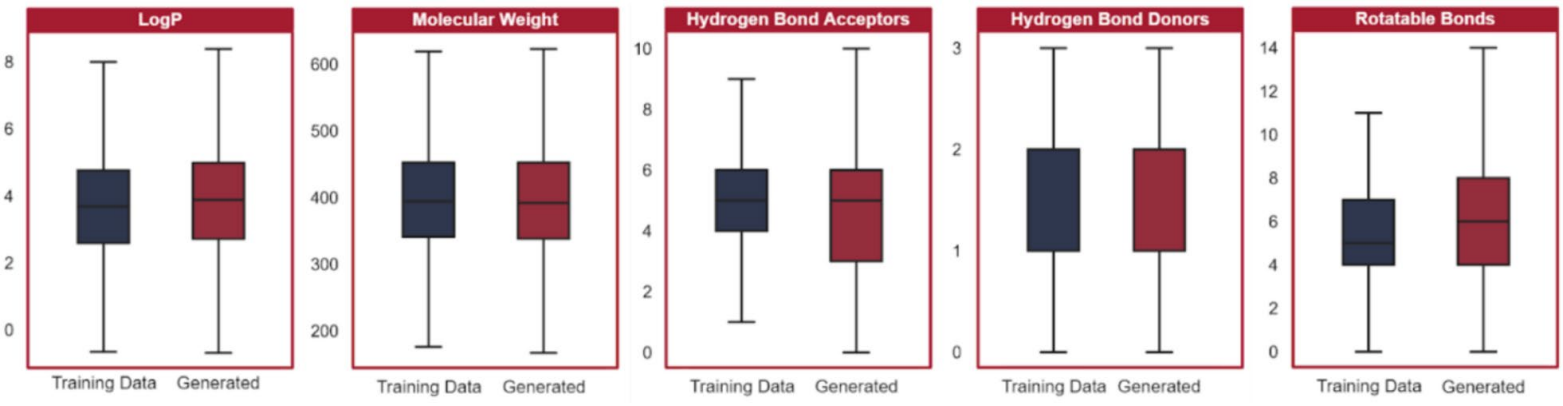
Distributions of molecular descriptors from the training data and the generated data. The distributions of the individual molecular descriptor values overlap significantly between the molecules in the training data and the molecules generated by the LSTM. The median and lower quartile values are equal in the distribution of hydrogen bond donors; thus, there is no additional median mark

Molecules were evaluated based on the five properties of the (modified) RO3. We compared the molecules generated by the LSTM to those in the original training data to ensure the model was operating in the same chemical space. We applied principal component analysis (PCA) to visualize the five properties, as shown in Fig. 4, and additionally visualized all five properties individually as shown in Fig. 5. The properties of the molecules in the original dataset and the molecules generated by the LSTM overlap significantly, indicating the model’s ability to accurately recreate, but not directly copy, the training data.

In addition, we evaluated the structural diversity of the molecules generated by the LSTM. It is necessary to ensure a wide variety of molecules are created as drug candidates may fail in unexpected ways later in the drug development pipeline [42]. Generated molecules were represented as Morgan fingerprints, indicating structural properties of the molecule [43]. The Tanimoto similarity $T$ was then calculated for each pair of molecules ${\varvec{a}}$ and ${\varvec{b}}$, where $\left|{m}_{a}\cap {m}_{b}\right|$ is the total number of fingerprints in common and $\left|{m}_{a}\cup {\mathrm{m}}_{\mathrm{b}}\right|$ is the total number of fingerprints [44]:

$$T\left({\varvec{a}},{\varvec{b}}\right)=\frac{\left|{m}_{a}\cap {m}_{b}\right|}{\left|{m}_{a}\cup { m}_{b}\right|}$$

It follows that Tanimoto similarity varies between 0 and 1, with lower values implying more structural diversity. The mean Tanimoto similarity of 25,000 randomly selected molecules from the training data was 0.1572. The mean Tanimoto similarity between 500 randomly selected novel generated molecules was 0.1608, indicating comparable diversity.

## Molecule selection and fine-tuning

The best half of the molecules generated were selected by the nondominated sorting algorithm based on the five constraints outlined by the (modified) Rule of Three. In cases of a tie between molecules, random selection was used. This amounted to 4707 molecules selected as fine-tuning data from the original 9415. Selected molecules were fed into the LSTM, and this process of generation–selection–transfer was iterated on. A running list of the best molecules was kept and capped at 10,000 molecules to quicken convergence. These were considered along with the newly generated molecules by the nondominated sorting algorithm at each iteration.

## Optimization

**Fig. 6:**
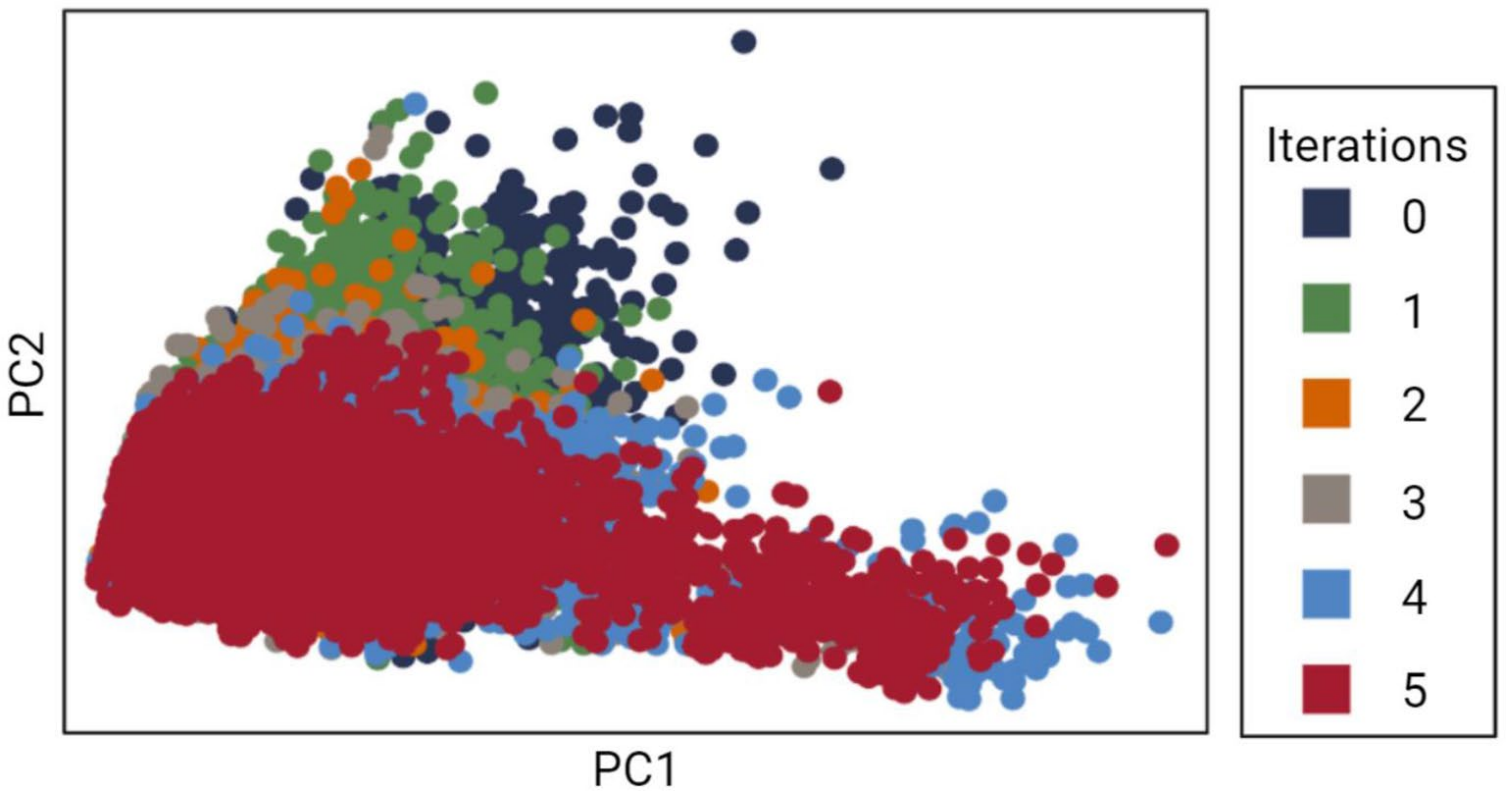
PCA projection of the molecular descriptors of generated molecules at each iteration of transfer learning**.** The model focuses on and begins to discover new regions of the chemical space to optimize the desired traits

**Fig. 7:**
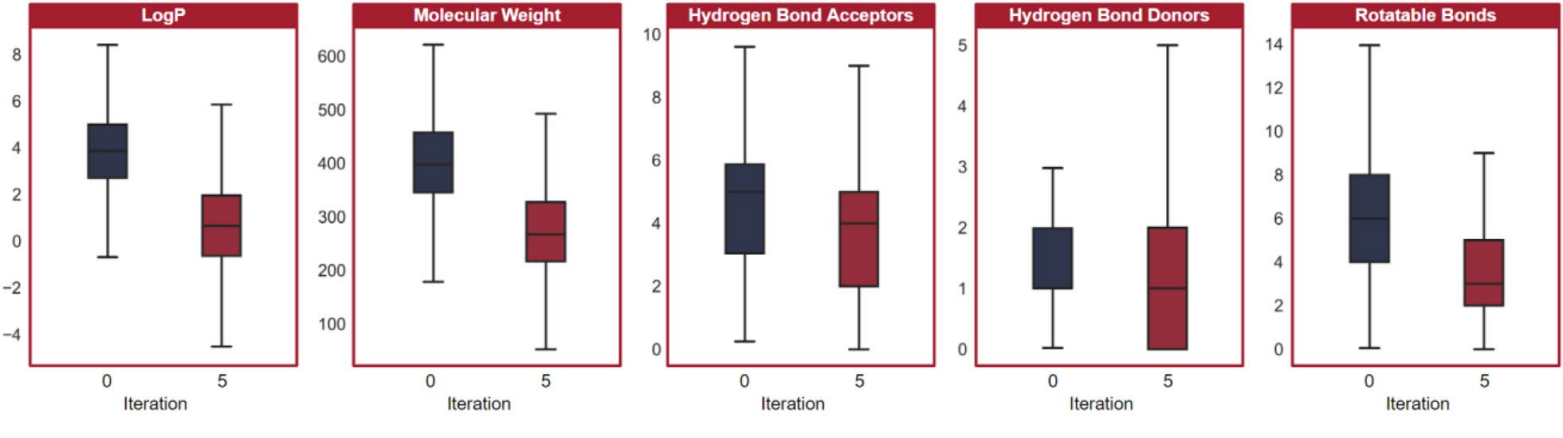
Distributions of molecular descriptors prior to transfer learning and after five iterations. All five descriptors measured were minimized as the LSTM model learns to generate more optimal molecules. The median and lower quartile values are equal in the distribution of hydrogen bond donors in the molecules generated prior to transfer learning; thus, there is no additional median mark. There is also no additional minimum mark in the distribution of hydrogen bond donors in the molecules generated after five iterations of transfer learning, as the lower quartile and minimum values are equal

Five iterations of transfer learning were run. We plotted the five properties measured onto two dimensions with PCA and compared the molecules generated at each iteration. As shown in Fig. 6, the LSTM focuses in on a more optimal section of the chemical space (Fig. 6). Additionally, we visualized the distributions of the five properties individually in Fig. 7. Molecules generated in the final iteration of transfer learning had minimized the objective values to levels far lower than molecules generated prior to transfer learning; thus, the model not only focuses in on, but also discovers new, more optimal areas of the chemical space.

**Fig. 8:**
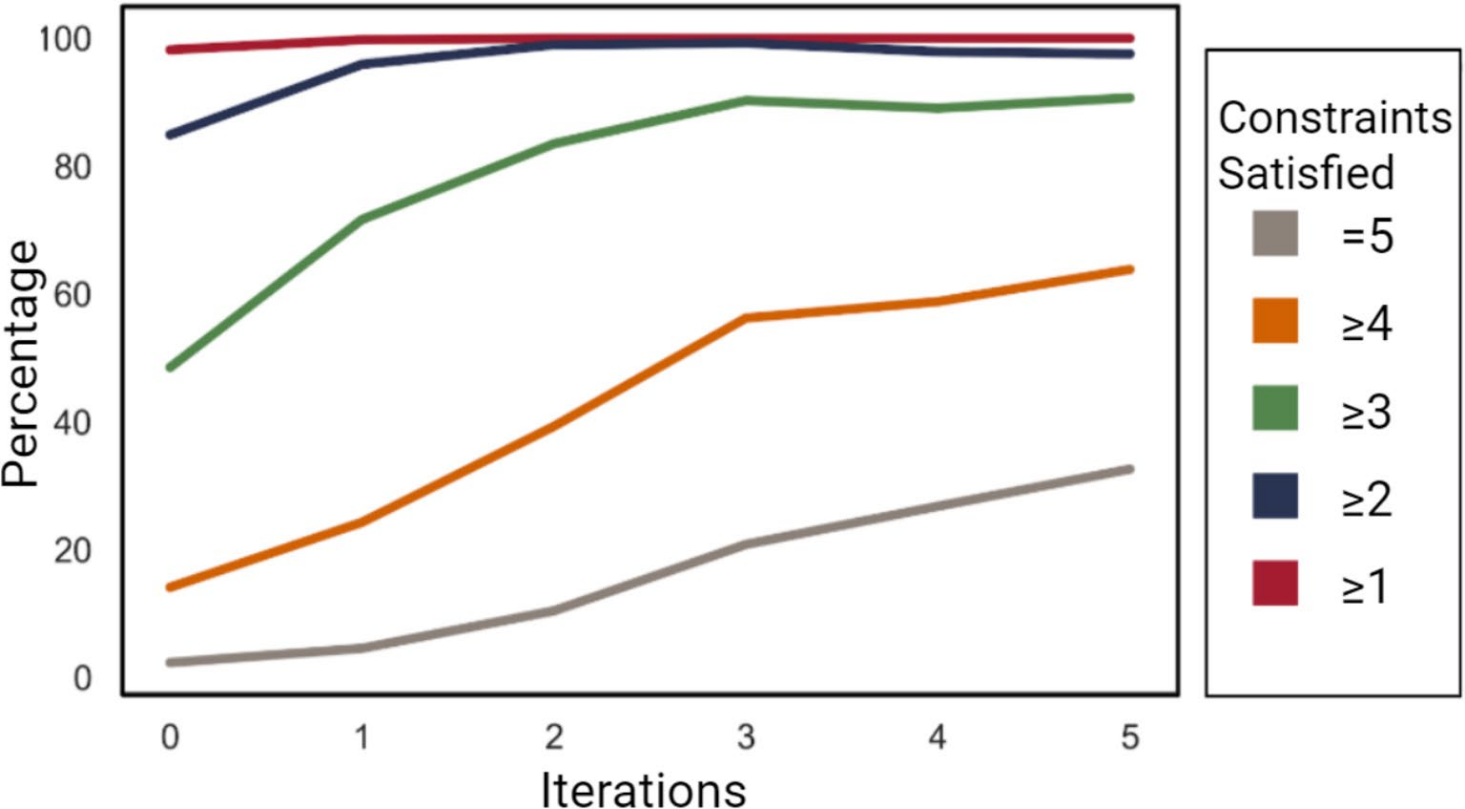
Percentage of molecules satisfying the constraints at each iteration**.** The percentage of molecules generated satisfying the constraints set by the (modified) Rule of Three were calculated. Significant improvement indicates the proposed algorithm is able to optimize multiple traits collectively

Examining the percentage of molecules that satisfied the constraints of the Rule of Three showed the model did indeed optimize the molecules. A nearly 14-fold increase was observed in the percentage of molecules satisfying all five constraints, as shown in Fig. 8 and Table 2.

**Table 2:** Percentages of generated molecules satisfying the constraints at each iteration of transfer learning

Iterations	Number of constraints satisfied				
	≥ 1 constraints (%)	≥ 2 constraints (%)	≥ 3 constraints (%)	≥ 4 constraints (%)	≥ 5 constraints (%)
0	98.20	84.92	48.53	14.14	2.36
1	99.79	95.94	71.67	24.31	4.59
2	99.99	99.00	83.52	39.33	10.46
3	99.99	99.27	90.28	56.25	20.85
4	100	97.92	89.08	58.82	26.82
5	100	97.58	90.70	63.85	32.62

In addition to optimizing the properties measured, the LSTM improved in both the accuracy and structural diversity of the molecules it generated. Prior to transfer learning, 77% of the molecules generated were valid; after five iterations, 86% were valid. The Tanimoto similarity of 500 randomly selected generated molecules decreased to 0.1218 from 0.1608 over the five iterations. This may be attributed to the molecule-size dependence of the Tanimoto metric [44] and the expanded chemical space in which the LSTM is operating in.

**Fig. 9:**
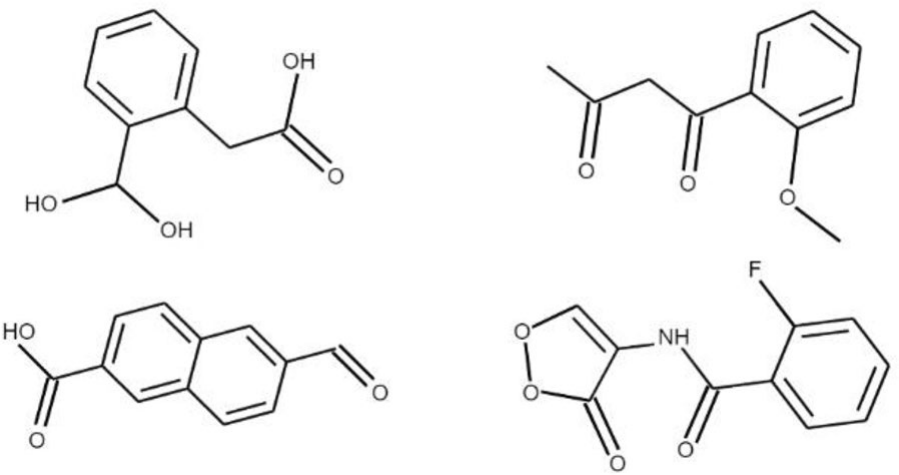
Randomly selected molecules generated in the final iteration of transfer learning

A few molecules were randomly selected from those generated in the final iteration of transfer learning and depicted in Fig. 9.

## Conclusion

In this work, we applied a recurrent neural network in conjunction with a nondominated sorting algorithm to create a cycle for multiobjective de novo drug design. Initially, the long short term memory (LSTM) recurrent neural network was able to generate new molecules with similar properties and similar diversity to the original training data. We then applied a nondominated sorting algorithm to select the best of the molecules generated. Five properties stemming from the Rule of Three were considered as a proof of concept, and the LSTM was iteratively fine-tuned on the molecules selected. Significant improvement was observed in the molecules generated across all properties measured, showing the multiobjective ability of the cycle proposed.

We outline three primary benefits of the proposed approach. This cycle of de novo drug design uniquely combines scalable generation of molecules with multiobjective optimization. Additionally, large quantities of data are not required to train the model, as it generates its own data as it trains. Finally, our system does not rely on any scoring functions. This makes for more accurate optimization and easy extension onto other molecular properties. The nondominated sorting algorithm still has downsides however. In particular, unrealistic or inferior molecules may seem worthy as one good property can carry it through selection. This problem may be mitigated in future work by adopting hard filters or removing outliers during the training process.

Additional improvements to this method can be made through the use of more elaborate selection mechanisms. Factoring in crowding distance (diversity) in the nondominated sorting algorithm may produce an even wider array of molecules. Other techniques of data preprocessing (e.g., encodings, paddings) may increase efficiency and accuracy, reducing the number of duplicates and invalid molecules generated. Most importantly, optimizing for more properties, specifically activity on a target, would further validate the efficacy of the proposed method and make it more applicable in industry.

De novo drug design is slowly making its way into drug development pipelines throughout the world. A multiobjective system such as the one proposed would be able to better the quality of molecules coming out of early stage drug discovery, complementing methods currently in use. Further exploration of machine learning in drug discovery provides enormous potential to reduce the cost and time associated with the development of drugs.

## Data Availability

All data used in this work is provided at https://github.com/jyasonik/MoleculeMO. SMILES data was extracted from the open source ChEMBL dataset.
